# EEG-Brain Activity Monitoring and Predictive Analysis of Signals Using Artificial Neural Networks

**DOI:** 10.3390/s20123346

**Published:** 2020-06-12

**Authors:** Raluca Maria Aileni, Sever Pasca, Adriana Florescu

**Affiliations:** Department of Applied Electronics and Information Engineering, Faculty of Electronics, Telecommunications and Information Technology, Politehnica University of Bucharest, 060042 Bucharest, Romania; pasca@colel.pub.ro (S.P.); adriana.florescu@upb.ro (A.F.)

**Keywords:** EEG, PPG, EMG, epilepsy, signal processing, brain monitoring, artificial neural network, predictive analysis

## Abstract

Predictive observation and real-time analysis of the values of biomedical signals and automatic detection of epileptic seizures before onset are beneficial for the development of warning systems for patients because the patient, once informed that an epilepsy seizure is about to start, can take safety measures in useful time. In this article, Daubechies discrete wavelet transform (DWT) was used, coupled with analysis of the correlations between biomedical signals that measure the electrical activity in the brain by electroencephalogram (EEG), electrical currents generated in muscles by electromyogram (EMG), and heart rate monitoring by photoplethysmography (PPG). In addition, we used artificial neural networks (ANN) for automatic detection of epileptic seizures before onset. We analyzed 30 EEG recordings 10 min before a seizure and during the seizure for 30 patients with epilepsy. In this work, we investigated the ANN dimensions of 10, 50, 100, and 150 neurons, and we found that using an ANN with 150 neurons generates an excellent performance in comparison to a 10-neuron-based ANN. However, this analyzes requests in an increased amount of time in comparison with an ANN with a lower neuron number. For real-time monitoring, the neurons number should be correlated with the response time and power consumption used in wearable devices.

## 1. Introduction

### 1.1. Aim of the Work

Epilepsy is a disease that affects about 1% of the world’s population. Crisis identification involves multi-channel EEG monitoring for 24–72 h. Crisis detection (grand mal) is essential for the diagnosis of epilepsy, the control of the crisis, and warning the patients in sufficient time before the seizure. Moreover, the observation and investigation of the biomedical signals values before an epileptic seizure are beneficial for developing prevention and support systems for patients because by informing the patient that an epilepsy seizure is about to occur, he or she can take his or her safety measures promptly. The correlations and covariance between the biomedical signals that EEG, PPG, and EMG collect from sensors are essential because, in the case of the patients with epilepsy, the heart rate increases, and uncontrolled tremors of the muscles or their stiffening may occur.

In the case of the patients with epilepsy, the real-time monitoring based on wearable devices with EEG, PPG, EMG integrated, and biomedical signals predictive analysis based on neural network systems with reduced processing time and low power computing is essential for warning before a seizure, and patients fall prevention.

### 1.2. State-of-the-Art

There are scientific studies that specify the use of artificial intelligence, using methods such as deep neural networks for patients’ ECG-based authentication [1], ResNet-based signal recognition [2], arrhythmia detection [3,4], or learning feed-forward and recurrent neural networks [5]. The automatic signal detection was used in studies based on the discrete wavelet transform (DWT) for automated detection [4] or automated heartbeat classification [6].

The electrical activity of the brain monitoring by EEG (electroencephalogram) is useful to study the disease pathologies by analyzing the numerical distribution of data and correlating the brain signals (EEG) with other types of biomedical signals such as electrical activity of the heart obtained by electrocardiogram (ECG), heart rate monitoring by photoplethysmograph (PPG), and electrical activity produced by muscles by electromyography (EMG) [7,8,9]. 

To analyze the pathology of chronic diseases, the researchers also used the multivariate analysis of EEG, ECG, and PPG signals [10,11].

Mainly for predictive analysis of influence factors that generate a pathology or of the biomedical signal changes that could anticipate the existence of pathology are software applications for signal acquisition from sensors (EEG, ECG, PPG, or EMG), correlations [12], univariate, bivariate [13,14,15] or multivariate analyzes [16,17] of numerical data used, but computational methods [18] based on mathematical models are also used. Thus, computational models use studies on large populations (e.g., 274 patients [19]) and a large volume of data (e.g., 183 seizures recorded in 3565 h [20]). These analyses aim to find valid patterns [21] for a large population with similar independent variables (age, gender).

For the prediction of epileptic seizures, researchers used technologies such as machine learning, data mining, artificial neural networks [22] (backpropagation algorithm-for recognition and classification of EEG signals [23,24]), fuzzy systems [25], and predictive analysis statistics (multivariate [26], bivariate or univariate).

The study of the correlations between various electrical signals captured (e.g., EEG, ECG, PPG, and EMG) from the human body is essential because, in the case of patients with neurological disorders, the phenomenon of comorbidity exists and consists of overlapping of several diseases.

The electroencephalogram (EEG) represents a set of fluctuating field potentials produced by the simultaneous activity of a large number of neurons [27] and captured by electrodes located on the scalp. The EEG system consists of 10–20 metal electrodes distributed on the skin surface of the head and connected by 36 wires to the recording device. It measures the electrical potential detected by each electrode. EEG can be used in monitoring the brain during anesthesia [28], surgical procedures [29], and investigations of brain disorders (psychoses [30], meningoencephalitis [31], Parkinson [9], Alzheimer [32,33,34,35,36,37], dementia [38], epilepsy [39,40,41,42], central motor neuron syndrome [43], cerebral palsy [44,45,46], and muscular dystrophy [47]). Mainly, EEG systems are used to diagnose and monitor patients with neuropathology, especially in diagnosis of epilepsy and in studying the seizures, as well as the monitoring of treatment and evolution.

Electroencephalographic reactivity is evaluated using simple tests: eye-opening, hyperpnea (slow and full breathing), and intermittent light stimulation obtained with short and intense light discharges with gradually increasing frequency. The EEG assessment takes approximately 20 min and does not require hospitalization [48].

In the case of an electroencephalogram, the risks are minimal. Still, intermittent light stimulation or hyperventilation can produce epileptic seizures. Therefore, the examination is performed under the supervision of a physician who can recognize the crisis and immediately establish appropriate safety and therapeutic measures.

Epilepsy is a chronic disease of the brain that manifests through partial (focal) or generalized seizures due to spontaneous electrical discharges that occur in the brain.

Manifestations consist of involuntary movements of different body segments and abnormal neuro-vegetative sensations in the body. EEG analysis can be used to diagnose and monitor the patient in various stages of the disease (focal or generalized seizures, sleep) [38,39,40,41].

### 1.3. Contribution

In this paper, we present an efficient method for the detection of seizures based on artificial neural networks and correlations between biomedical signals. 

Our study included 30 subjects from the CAP Sleep Database [49,50]. Our selected records were sampled at 160 Hz. The records consisted of both normal EEG and EEG spikes specific to epileptic seizures. The signals captured were from 13 EEG channels, submentalis and bilateral anterior tibialis EMG, and an earlobe PPG sensor. We used the artificial neural network and the Levenberg–Marquardt backpropagation optimization algorithm in MATLAB for implementing the classification and 3D plots. Data pre-processing and feature extraction were implemented using MATLAB 2019a (Mathworks, Santa Clara, CA, USA). All the experiments were carried out in Windows 8.1, 8 GB RAM, and 64-bit operating system.

The rest of the paper is structured as follows: the methods for signals decomposition, filtering, EEG biomedical signals, and theoretical methodology are presented in Section 2. Section 3 presents the predictive analysis of the signals using artificial neural networks. Aspects concerning the biomedical signals covariance are discussed in Section 4. The conclusions of the work are presented in Section 5.

## 2. Materials and Methods

The proposed method was tested using the CAP Sleep Database. The CAP Sleep Database comprises 40 recordings of patients (male and female) diagnosed with nocturnal frontal lobe epilepsy. The record duration is 8 h, approximately.

Our study included 30 subjects from the CAP Sleep Database. Our selected records were sampled at 160 Hz. The records consist of both normal EEG and EEG spikes specific to epileptic seizures. We analyzed 30 EEG recordings 10 min before a seizure and during the seizure in 30 patients with epilepsy. The signals analyzed are from 13 EEG channels, submentalis and bilateral anterior tibialis EMG, and an earlobe PPG sensor.

Within this research, the topic has used the detection of electrical signals from the brain using the EEG head with non-invasive electrodes (for the available biomedical signals in the PhysioNet databases). 

In the discrete-time domain, digital filters (low-pass filter for signals with a frequency lower than a selected cutoff frequency and a high-pass filter that passes signals with a frequency higher than a cutoff frequency chosen) have been used for signal analysis. 

**Figure 1:**
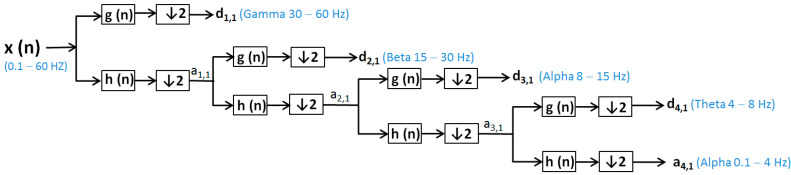
Electroencephalogram (EEG) from a patient with no seizure-signal filtering and decomposition using the discrete wavelet transform (DWT) method.

**Figure 2:**
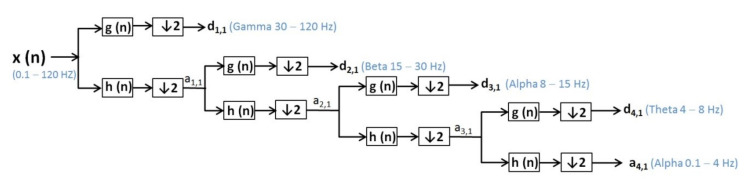
EEG from a patient with epileptic seizure-signal filtering and decomposition using the DWT method.

Discrete wavelet transformation (DWT) [48] is calculated by additional high-pass and successive low-pass filters and sub-sampling using the Mallat algorithm [51]. Additional filtering applied to a real EEG signal leads to double the number of data from the original one being requested after each filtration to reduce the number of samples by sub-sampling of the EEG signal. DWT uses the dyadic variant. In the wavelet analysis, approximations (a (n)) and details (d (n)) are used (Figure 1 and Figure 2):

1. Approximations (a (n)) are the components at high scales and low frequencies;
2. Details (d (n)) are components at low levels and high rates.

To reduce the continuous-time signal to a discrete-time signal, the EEG signals were sampled with a sampling frequency (*f*s = 160 Hz). EEG signals were filtered by a low-pass filter (60 Hz) and a high-pass filter (0.1 Hz) and decomposed using the discrete wavelet transform [52,53] for patients without epilepsy (Figure 1). 

In the case of epilepsy, seizures detection consists of finding EEG segments with seizures and onset and offset points [53]. For pattern profiling, it is necessary to monitor a large population of patients with epilepsy for 24–48 h. Because gamma frequency oscillations (30–120 Hz) often precede interictal epileptiform spike discharges (IEDs) [54], we used DWT with Daubechies function, and we considered the low-pass filter 120 Hz to observe the gamma wave specific to an epileptic seizure. Some scientific papers report the values around 100–600 Hz for gamma waves—that is, not associated with IEDs, but occurring during epileptic seizures [54,55]. However, other researchers [54,56] reported the fluctuation of gamma wave values.

EEG signals were filtered by a low-pass filter (120 Hz) and a high-pass filter (0.1 Hz) and decomposed using the discrete wavelet transform for patients with epileptic seizures (Figure 2).

x (n)—signal (0.1–60 Hz), respective for patient with seizure x (n)—signal (0.1–120 Hz);
h(n)—low-pass filter (LPF);
g (n)—high-pass filter (HPF);
d (n)—the signal of the detail produced by HPF, e.g., d1, 1, d2, 1, d3, 1, d4, 1;
a (n)—the signal produced by LPF, is a rough approximation, e.g., a1, 1, a2, 1, a3, 1, a4, 1;
↓2—down sampling by two.

**Figure 3:**
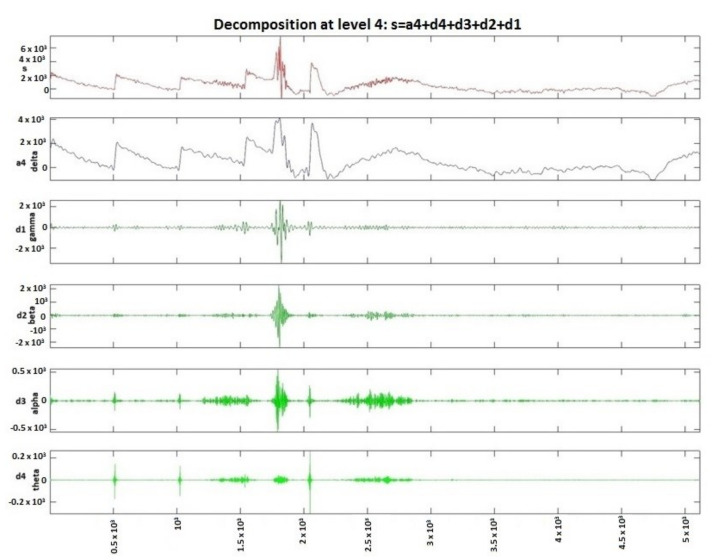
Patient before and after the seizure, signal decomposition on four levels using DWT.

The wavelet transform is a way to implement a particular type of signal representation called multi-resolution analysis [57,58]. The analyzed signal is described by a succession of details and approximations that contain more information. Each level of approximation (Figure 1 and Figure 2) contains information available at the previous level, which is an added component of detail. In Figure 3, the signal processed by discrete wavelet transform and Daubechies method using four decomposition levels for a patient before and after a short seizure is presented. In Figure 3, detail d1 represents gamma waves, detail d2 represents beta waves, detail d3 represents alpha waves, detail d4 represents theta waves, and the approximation a4 represents delta waves. 

**Figure 4:**
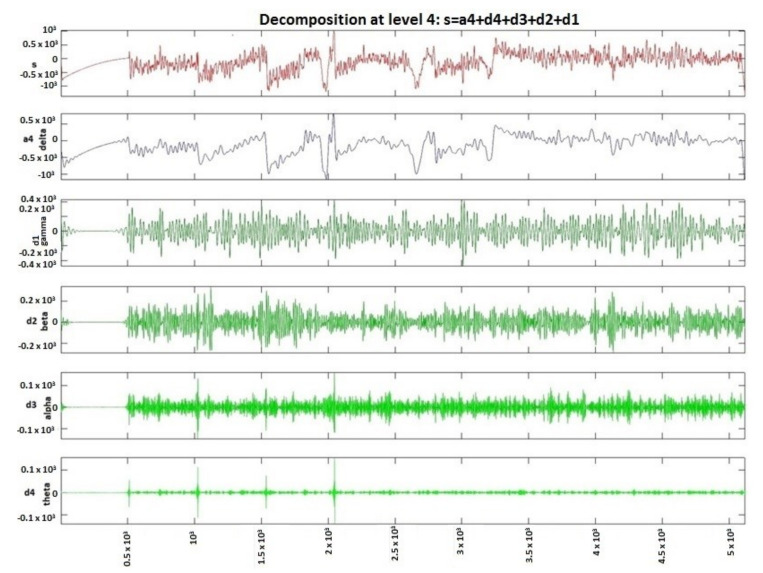
Patient with epileptic seizure, signal decomposition on four levels using DWT.

In Figure 4, the signal processed by discrete wavelet transform and Daubechies method using four decomposition levels for a patient with epileptic seizures is presented. In Figure 4, the detail d1 represents gamma waves, detail d2 represents beta waves, detail d3 represents alpha waves, detail d4 represents theta waves, and the approximation a4 represents delta waves. From Figure 4, it is evident that the presence of the gamma waves with values equal to or greater than 120 shows that a seizure phase is present. Moreover, the epileptic spikes are very evident in Figure 4.

**Figure 5:**
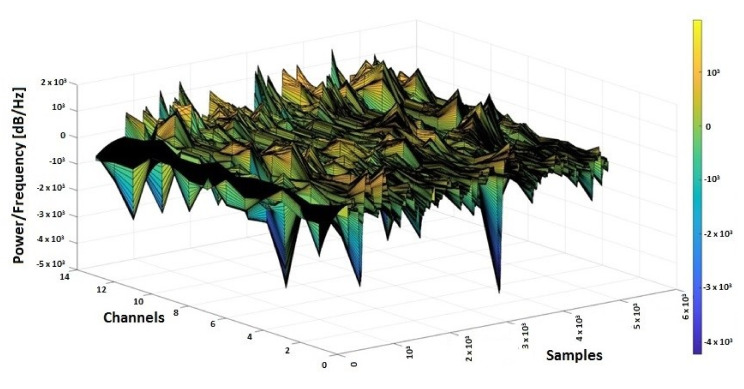
3D spectrogram of EEG signals from 13 channels.

In Figure 5, the 3D spectrogram of the signals from all 13 channels of electro-cap used for monitoring a patient with an epileptic seizure is presented. The epileptic gamma waves spikes (with the yellow-red color market on the graphic) that are over 200 or 400, indicating abnormal frequencies for gamma waves that occur on seizures, are also evident from Figure 5.

## 3. Biomedical Signal Selection

To analyze the correlation and covariance between signals, signals such as EEG (related to the frontal lobes FP1-F3, FP2-F4), EMG, and PPG from a patient n1 with no epileptic seizures and a patient n2 with epileptic seizures were selected. 

**Figure 6:**
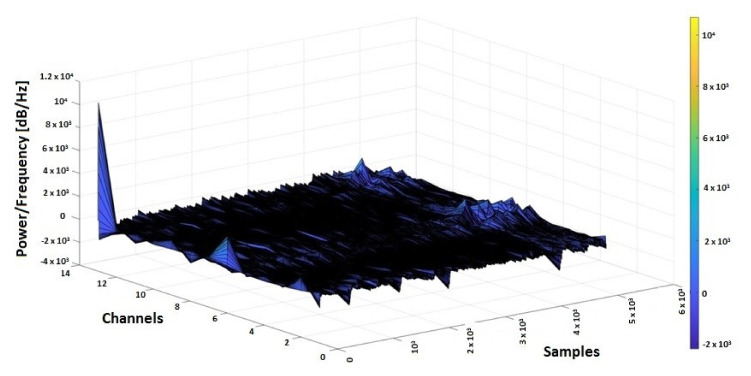
3D spectrogram of signals EEG from 13 channels for patient n1 with no epileptic seizures.

**Figure 7:**
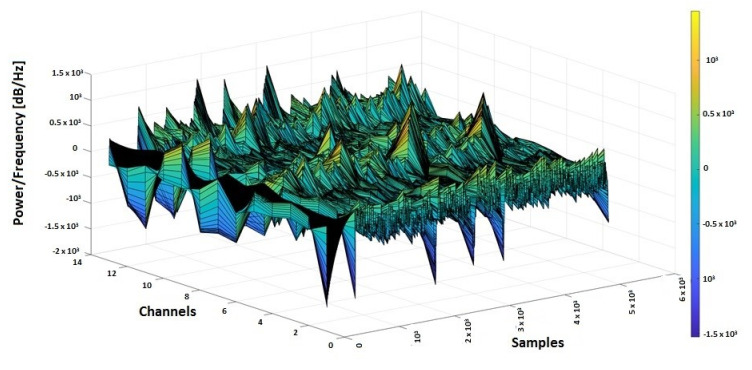
3D spectrogram signals EEG from 13 channels for patient n2 with epileptic seizures.

The purpose of using PPG and EMG signals in correlation with EEG was to find a modification of the biomedical signals collected from wearable devices that could anticipate an epilepsy seizure and to use a software system to send medical alerts in advance [59,60,61,62,63,64,65]. From the CAP Sleep Database, the biomedical signals taken from 2 patients (n1 and n2) were used for the actual study. In Figure 6 and Figure 7, the 3D spectrograms for the EEG signals (Fp2-F4, F4-C4, C4-P4, P4-O2, F8-T4, T4-T6, FP1-F3, F3-C3, C3 -P3, P3-O1, F7-T3, T3-T5, C4-A1) taken from patients n1 and n2 are presented. In the case of patient n1, the epileptic spikes for gamma waves cannot be observed (Figure 6), but in the case of patient n2, these spikes are evident, marked with yellow-orange in the 3D spectrogram (Figure 7) and being above the 120 Hz threshold.

## 4. Results Based on Predictive Analysis of the Signals Using Artificial Neural Networks

For predictive analysis of EEG signals, artificial feed-forward neural networks are used based on the Levenberg–Marquardt backpropagation optimization algorithm.

The functional units within the neural networks consisted of:

- Input units represented by the values of the EEG matrix for patients with epilepsy seizures.

Hidden groups (data) given by the number of neurons (10, 50, 100, and 150 neurons, respectively).

- Outputs are represented by the values of the EEG matrix for patients who do not have seizures.

For optimization, the Levenberg–Marquardt algorithm was used, which approximates the Hessian matrix (*H*) as follows (1):

(1) $$H=J^{T}J$$

where:

- *J* is the Jacobian matrix containing the derivatives of the error function concerning weights (w) and biases (b);
- *J^T^* is the transposed Jacobian matrix;
- *e* is the vector of errors.

The Levenberg–Marquardt algorithm uses the following parameter updating rule (Equation (2)):

(2) $$x_{k+1}=x_{k}-{\left[J^{T}J+\mu I\right]}^{-1}J^{T}e$$

**Figure 8:**
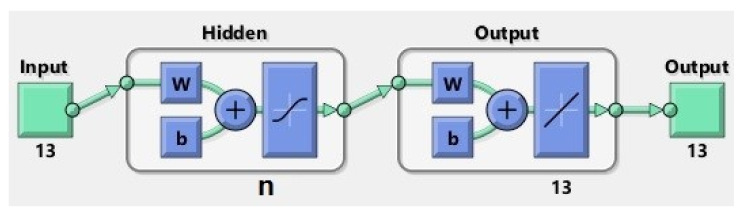
Artificial neural network (ANN) with *n* neurons, *n* ∈ {10, 50, 100, 150}.

For this purpose, four neural networks were designed with *n* hidden neurons (Figure 8), where $n\in \left\{10,\;50,\;100,\;150\right\}$, to estimate the occurrence of epilepsy seizures, compared with EEG signals taken from a healthy patient, respectively, with EEG signals received from the patient with no seizures. The artificial neural network (ANN) architecture models (with 10, 50, 100, and 150 respective hidden neurons) used for the prediction of the epileptic seizures have a two-layer feed-forward network with hidden sigmoid neurons and linear output neurons, and allow the training and evaluation of the performance using mean square error (MSE) and regression analysis (R). The proposed ANNs structures are based on the principal elements:

- input data (matrix 13 × 5120 samples);
- hidden layer with *n* neurons, *n* ∈ {10, 50, 100, 150};
- output (target) data (matrix 13 × 5120 samples);
- train set (70% of samples) that is used to provide an independent measure of network performance during and after training;
- test set (15% of samples) that is used during training, and the network is adjusted according to its error;
- validation set (15% of samples) is used to measure network generalization, and to halt training when generalization stops improving.

In Table 1, the principal parameters for ANNs with 10, 50, 100, and 150 neurons are presented.

Prediction and optimization were made with a feed-forward backpropagation multi-layer neural network.

The input data-independent variables (matrix input) *X*_1_ = EEG signal (EEG3) taken when the patient does not have seizures.

The target data-dependent variables (matrix target) *Y*_1_ = EEG signal (EEG1) taken from a patient with epilepsy. The target (*Y*_1_) represents the desired output for the given input, *X*_1_. We consider the real output matrix (*D*).

**Figure 9:**
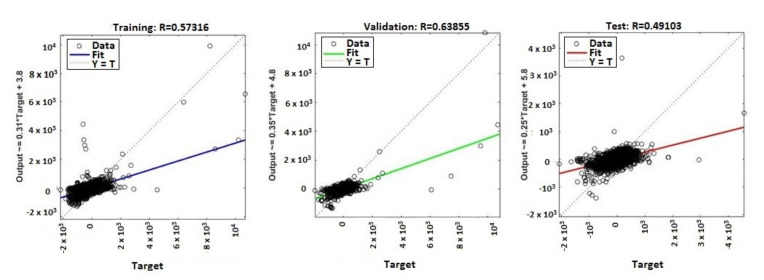
Regression (*R*^2^) for validation, test, and training—ANN with ten neurons.

**Figure 10:**
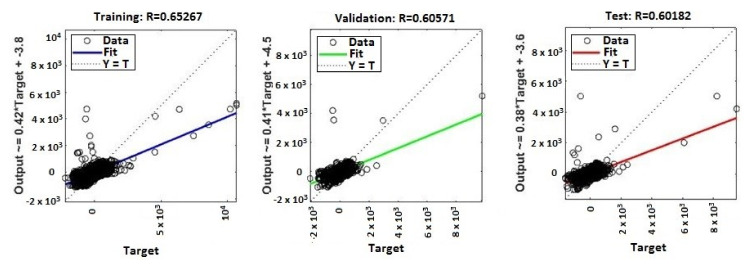
Regression (*R*^2^) for validation, test, and training—ANN with 50 neurons.

**Figure 11:**
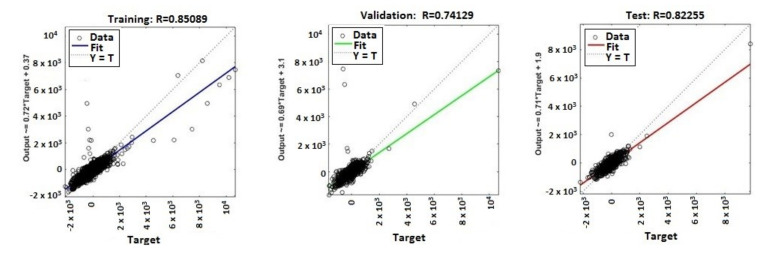
Regression (*R*^2^) for validation, test, and training—ANN with 100 neurons.

**Figure 12:**
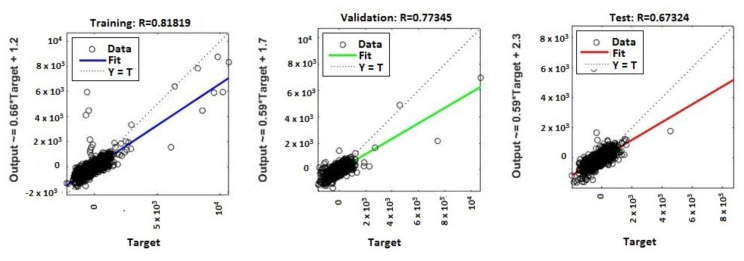
Regression (*R*^2^) for validation, test, and training—ANN with 150 neurons.

The continuous training of neural networks is based on extensive datasets; 70% (3584 samples) of the total data generated by the ANNs were used to train the model, while 15% (768 samples) of the data was used for testing and 15% (768 samples) for validation (Figure 9, Figure 10, Figure 11 and Figure 12). Regression analysis of the ANN model showed the *R*^2^ (regression) values for training between 0.57316 for the ANN with ten neurons, 0.65267 for the ANN with 50 neurons, 0.85089 for the ANN with 100 neurons, and 0.81819 for the ANN with 150 neurons, showing the higher accuracy and significance of the ANN model for the ANN with 100 neurons, respective to the ANN with 150 neurons.

MATLAB libraries were used to perform the functions and the code sequences within the neural networks. The regression plots (9–12) show a regression between network outputs and network targets. The parameterized linear regression model is given by mathematical relation (3). The *R* (Equation (3)) value indicates the relationship between the outputs (*y*) and targets. If *R* = 1, this indicates that there is an exact linear relationship between outputs and targets. If the *R*-value is close to zero, then there is no linear relationship between the outputs and targets.

(3) $$R=D=\sum_{j=1}^{M}w_{j}x_{j}+\varepsilon \;\;\;\Leftrightarrow \;R=w^{T}x+\varepsilon$$

where:

- *ε* is the error;
- *w_j_* is synaptic weight;
- *x* is the input matrix;
- *M* is the model order;
- *T* denotes matrix transposition (Equations (4) and (5)).

(4) $$w={\left[w_{1},w_{2},\ldots ,w_{M}\right]}^{T}$$

(5) $$x={\left[x_{1},x_{2},\ldots ,x_{M}\right]}^{T}$$

From the regression graphs for testing, training, and validation for neural networks with 10, 50, 100, and 150 neurons (Figure 9, Figure 10, Figure 11 and Figure 12), and from values presented on Table 1, it is evident that the value of the *R* regression for training, validation, and testing is in a direct relationship with the number of neurons of the network. The regression value *R* close to zero indicates that is no linear relationship between outputs and targets. Moreover, if *R* is very close to 1, it shows a good match and an exact linear relationship between the outputs and targets. From the regression graphs, it is observed that the value of the regression for test, training, and validation is close to the value 1, which indicates a good match between inputs, outputs, and objectives. From Figure 9, it can be observed that, in the case of the neural network with ten hidden neurons, the values of the regression for test, validation, and training are in the inequality report *R_Test_* < *R_Training_* < *R_Validation_*, the regression is lower than 1, and the higher one is the regression for validation (*R_Validation_* = 0.63855). From Figure 10, we observed that, in the case of the neural network with 50 hidden neurons, the values of the regression for test, validation, and training are in the inequality report *R_Test_* < *R_Validation_* < *R_Training_*, the regression is lower than 1, and the higher one is the regression for training (*R_Training_* = 0.65267). In Figure 11, it can be observed that, in the case of the neural network with 100 hidden neurons, the values of the regression for test, validation, and training are in the inequality report *R_Validation_* < *R_Test_* < *R_Training_*, the regression is lower than 1, and the higher one is the regression for training (*R_Training_* = 0.85089). From Figure 12, it is evident that, in the case of the neural network with 150 hidden neurons, the values of the regression for test, validation, and training are in the inequality report *R_Test_* < *R_Validation_* < *R_Training_*, the regression is lower than 1, and the higher one is the regression for training (*R_Training_* = 0.81819).

**Figure 13:**
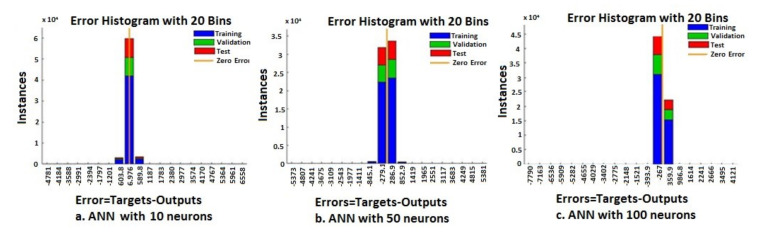
Error histograms—ANN with 10, 50, and 100 neurons.

**Figure 14:**
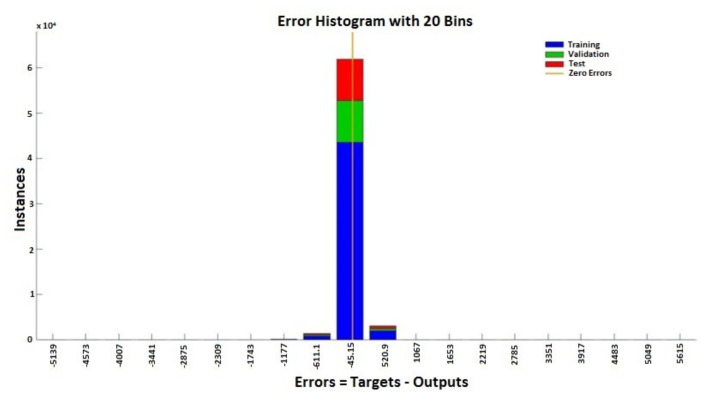
Error histogram—ANN with 150 neurons.

From the histograms of errors (Figure 13 and Figure 14), it can be observed that the increase in the number of neurons in the network leads to a decrease in the percentage of errors generated. The error histograms (Figure 13 and Figure 14) show normal distributions with residuals (errors), indicating that many of the residuals fall on or near zero in the case of the ANN with 150 neurons. Analyzing Figure 13 and Figure 14, we can conclude that the ANN model with 150 neurons used for the prediction can generate an excellent prediction of epileptic seizures.

In Table 2 are presented for each neural network developed, the number of hidden neurons allocated, the processing time [seconds] of the neural network, and the values of the regression for training (*R_Training_*), test (*R_Test_*), and validation (*R_Validation_*). In Table 2, the processing time represents the total time allocated for training, test, and validation.

In the proposed ANN with *n* (10, 50, 100, and 150) neurons, we defined the training set, test set, and validation set to check over-optimization. The validation set was used to measure network generalization, and to halt training when generalization stopped improving. The evaluation of the performance was done using mean square error (MSE) and regression analysis (R).

**Figure 15:**
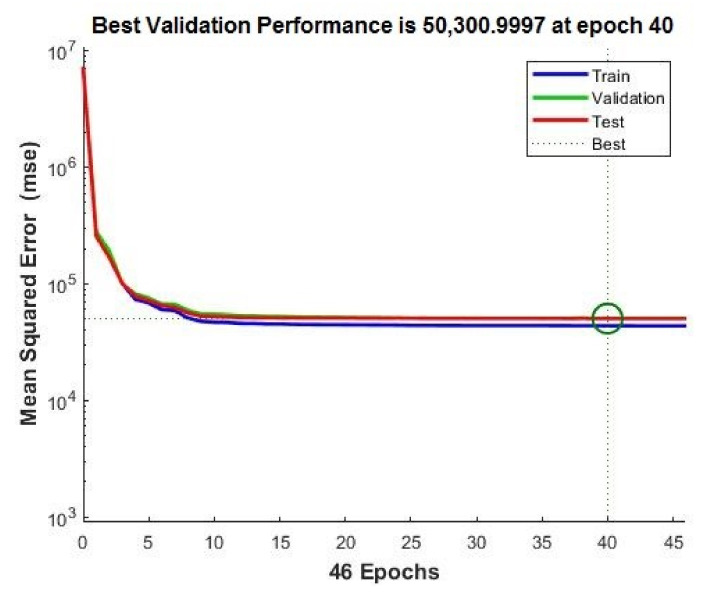
Neural network (10 neurons) best validation performance.

**Figure 16:**
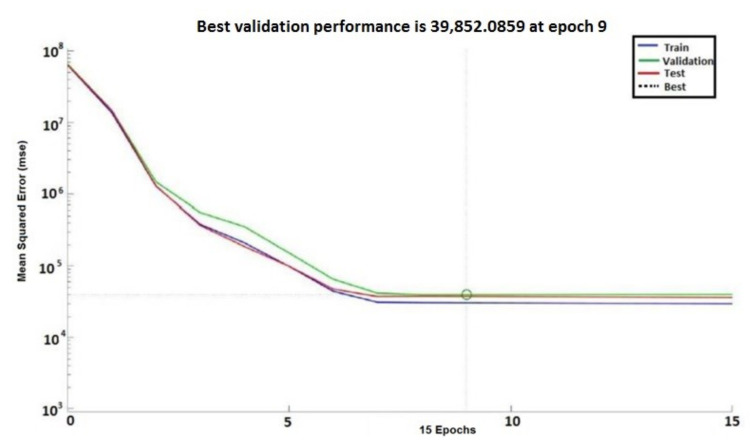
Neural network (150 neurons) best validation performance.

In Figure 15 and Figure 16, the performances of the neural networks with 10 and 150 neurons, respectively, are presented. In Figure 15 and Figure 16, error vs. epoch is plotted for the validation. The best validation is taken from the epoch with the lowest validation error. On the *y* axis of the charts, the mean squared error (MSE) (Equation (6)) is presented. The best validation is taken from the epoch with the lowest validation error. Mainly, the error reduces after more epochs of training.

(6) $$\mathrm{MSE}SE=\frac{1}{n}\overset{n}{\underset{i=1}{\sum }}{\left(y_{i}-{\hat{y}}_{i}\right)}^{2}$$

where:

$y_{i}$ is the vector of observed values;
${\hat{y}}_{i}$ is the vector of predicted values.

However, the best validation performance was generated in 40 epochs, whereas 47 epochs were run to confirm the model accuracy for the ANN with ten neurons (Figure 15). The best validation performance was generated in 9 epochs, whereas 15 epochs were run to confirm the model accuracy for the ANN with 150 neurons (Figure 16). In comparison with the ANN with ten neurons, the ANN with 150 neurons shows higher performance.

## 5. Discussion 

### 5.1. Biomedical Signals Covariance Analysis

In order to evaluate if the previously presented biomedical signals (EMG, PPG, and EEG) can be used to predict epileptic seizures, it is necessary to investigate the covariance between all the analyzed signals. Mainly, for two discrete signals, *x*(*k*) and *y*(*k*), correlation is a discrete function in time (Equation (7)), defined by:

(7) $$r_{xy}\left(k\right)=\overset{+\infty }{\underset{n=-\infty }{\sum }}x\left(n\right)y\left(n-k\right)$$

where *k* = 0, 1, 2, ….

Using the correlation function of two signals, the similarity between the signals can be appreciated. The autocorrelation function has a maximum in origin when *k* = 0 and can be used to determine the periodicity of real signals. The autocorrelation function (Equation (8)) is defined by:

(8) $$r_{xx}\left(k\right)=\overset{+\infty }{\underset{n=-\infty }{\sum }}x\left(n\right)y\left(n-k\right)$$

where: *k* = 0, 1, 2, ….

The signals EEG1 (no seizure) and EEG3 (with seizure) collected from patient n1, respective to the signals EEG2 (with seizure) and EEG4 (no seizure) collected from patient n2, were sampled at a rate of 160 Hz and filtered using high-pass (0.1 Hz) and low-pass filters (60 Hz for EEG with no seizure activity, respective to 120 Hz for EEG with seizure).

By analyzing the covariance matrix for EEG_i_, EEG_j_ (Equations (9), (11), (13), (15), (17) and (19)), and correlation coefficients (Equations (10), (12), (14), (16), (18) and (20)), we found that:

- between EEG1 and EEG3 is a negative covariance; this means that they are not in a linear dependence (Equation (9)). Because the correlation coefficient is negative (Equation (10)), it follows that EEG1 and EEG3 are in an inverse proportionality relationship.
- between EEG2 and EEG4 is a negative covariance, which means that EEG2 and EEG4 are not in a linear dependence (Equation (11)). Because the correlation coefficient is negative (Equation (12)), it follows that EEG1 and EEG3 are in an inverse proportionality relationship.
- between EEG1 and EEG4 is a positive covariance, which means that EEG1 and EEG4 are in a linear dependence (Equation (13)), and because the correlation coefficient is positive (Equation (14)), it follows that EEG1 and EEG4 are in a direct proportionality relationship.
- between EEG1 and EEG2 is a negative covariance (Equation (15)), which means that EEG1 and EEG2 are not in a linear dependence, and because the correlation coefficient is negative (Equation (16)), it follows that EEG1 and EEG2 are in an inverse proportionality relationship.
- between EEG2 and EEG3 is a positive covariance, which means that EEG2 and EEG3 are in a linear dependence (Equation (17)), and because the correlation coefficient is positive (Equation (18)), it follows that EEG1 and EEG4 are in a direct proportionality relationship.
- between EEG3 and EEG4 is a negative covariance (Equation (19)), which means that EEG3 and EEG4 are not in a linear dependence, and because the correlation coefficient is negative (Equation (20)), it follows that EEG3 and EEG4 are in an inverse proportionality relationship.

(9) $$cov\left(EEG1,\;EEG3\right)=1.0e+05*\left|\begin{array}{cc} 0.7206 & -0.0369\\ -0.0369 & 0.7206 \end{array}\right|$$

(10) $$R_{EEG1,\;EEG3}=\left|\begin{array}{cc} 1.0000 & -0.0272\\ -0.0272 & 1.0000 \end{array}\right|\Leftrightarrow r_{1,2}=r_{2,1}=-0.0272$$

(11) $$cov\left(EEG2,\;EEG4\right)=1.0e+05*\left|\begin{array}{cc} 0.5555 & -0.0900\\ -0.0900 & 5.7909 \end{array}\right|,$$

(12) $$R_{EEG2,EEG4}=\left|\begin{array}{cc} 1.0000 & -0.0502\\ -0.0502 & 1.0000 \end{array}\right|\Leftrightarrow r_{1,2}=r_{2,1}=-0.0502,$$

(13) $$cov\left(EEG1,\;EEG4\right)=1.0e+05*\left|\begin{array}{cc} 0.7206 & 0.0196\\ 0.0196 & 5.7909 \end{array}\right|,$$

(14) $$R_{EEG1,\;EEG4}=\left|\begin{array}{cc} 1.0000 & 0.0096\\ 0.0096 & 1.0000 \end{array}\right|\Leftrightarrow r_{1,2}=r_{2,1}=0.0096,$$

(15) $$cov\left(EEG1,\;EEG2\right)=1.0e+04*\left|\begin{array}{cc} 7.2061 & -0.5302\\ -0.5302 & 5.5551 \end{array}\right|,$$

(16) $$R_{EEG1,\;EEG2}=\left|\begin{array}{cc} 1.0000 & -0.0838\\ -0.0838 & 1.0000 \end{array}\right|\Leftrightarrow r_{1,2}=r_{2,1}=-0.0838,$$

(17) $$cov\left(EEG2,\;EEG3\right)=1.0e+05*\left|\begin{array}{cc} 0.5555 & 0.3163\\ 0.3163 & 2.5545 \end{array}\right|,$$

(18) $$R_{EEG2,EEG3}=\left|\begin{array}{cc} 1.0000 & 0.2655\\ 0.2655 & 1.0000 \end{array}\right|\Leftrightarrow r_{1,2}=r_{2,1}=0.2655,$$

(19) $$cov\left(EEG3,\;EEG4\right)=1.0e+05*\left|\begin{array}{cc} 2.5545 & -0.2580\\ -0.2580 & 5.7909 \end{array}\right|,$$

(20) $$R_{EEG3,\;EEG4}=\left|\begin{array}{cc} 1.0000 & -0.0671\\ -0.0671 & 1.0000 \end{array}\right|\Leftrightarrow r_{1,2}=r_{2,1}=-0.0671$$

**Figure 17:**
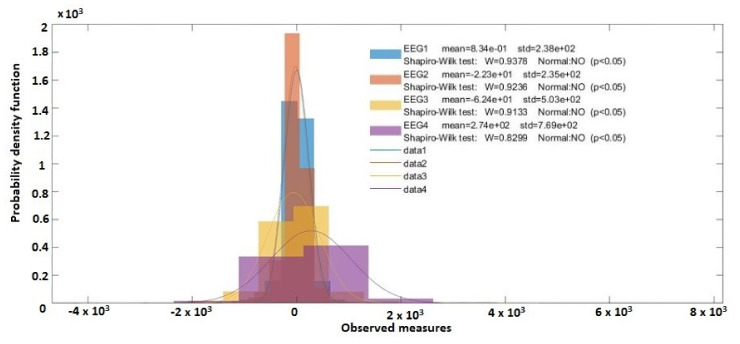
Distribution probabilities (Shapiro–Wilk test, Brainstorm).

Using the Shapiro–Wilk test (Figure 17) to evaluate the distribution of EEG1, EEG2, EEG3, and EEG4 signals in the Brainstorm application, it can be seen that the values for W_EEG1_ = 0.9378, W_EEG2_ = 0.9236, W_EEG3_ = 0.9133, and W_EEG4_ = 0.8299, are very close to 1, which means that the signals have a distribution close to the normal distribution.

The analysis of the covariances and correlations between EMG2 and EEG2 (Equations (21) and (22)) and PPG3 and EEG3 (Equations (23) and (24)), respective of those between EMG3 and EEG3 (Equations (25) and (26)), shows that there is a positive correlation and a direct covariance between signal pairs ((PPG3, EEG3) and (EMG3, EEG3)), respective of those between signal pairs (EMG2, EEG2), which could be exploited in anticipation of epilepsy seizures by predictive analysis using an ANN and a support decision system.

(21) $$cov\left(EMG2,EEG2\right)=1.0e+04*\left|\begin{array}{cc} 0.6864 & 0.0422\\ 0.0422 & 9.2137 \end{array}\right|,$$

(22) $$R_{EMG2,EEG2}=\left|\begin{array}{cc} 1.0000 & 0.0168\\ 0.0168 & 1.0000 \end{array}\right|\Leftrightarrow r_{1,2}=r_{2,1}=0.0168,$$

(23) $$cov\left(PPG3,\;EEG3\right)=1.0e+05*\left|\begin{array}{cc} 0.2690 & 0.0909\\ 0.0909 & 4.9304 \end{array}\right|,$$

(24) $$R_{PPG3,\;EEG3}=\left|\begin{array}{cc} 1.0000 & 0.0789\\ 0.0789 & 1.0000 \end{array}\right|\Leftrightarrow r_{1,2}=r_{2,1}=0.0789$$

(25) $$cov\left(EMG3,\;EEG3\right)=1.0e+05*\left|\begin{array}{cc} 2.1729 & 0.0122\\ 0.0122 & 4.9304 \end{array}\right|,$$

(26) $$R_{EMG3,EEG3}=\left|\begin{array}{cc} 1.0000 & 0.0037\\ 0.0037 & 1.0000 \end{array}\right|\Leftrightarrow r_{1,2}=r_{2,1}=0.0037,$$

In conclusion, the correlations and covariances between the biomedical signals (EEG, PPG, and EMG) collected from sensors are significant because, in the case of the patients with epilepsy, the heart rate increases and may generate uncontrolled tremors of the muscles or their stiffening. Furthermore, to patients having epilepsy, the comorbidity phenomena are present [66,67,68] and consist of overlapping of several diseases (diabetes, cardiovascular diseases, etc.) 

### 5.2. Comparative Analysis

To observe the performance of our proposed methodology, we compared our methods (DWT and ANN), validation, and accuracy of the results with the existing methods based on machine learning from the literature. Comparison is presented in Table 3, which contains the feature extraction methods, the machine learning methods, the validation methods, and also the classification accuracy.

### 5.3. Limitation and Future Scope

The proposed methods give significant results, but the ratio between best validation performance and processing time exhibits an inverse relationship and generates the limitation in real-time data processing because the neural network with 150 neurons has the best validation performance, but the increasing the number of neurons in the ANN generates an increase in the time required for data processing.

The other state-of-the-art methods do not analyze the problem of real-time processing through the perspective of the ratio between best performance validation and time.

However, an investigation for a new set of parameters and to learn algorithms to improve this is needed. Moreover, analyzing other physiological signals such as the heart’s electrical activity (ECG) along with EMG, PPG, and EEG may improve the investigations to detect biomedical parameters changes before or during the seizures.

## 6. Conclusions

In this work, we used artificial neural networks (ANN) for automatic detection of epileptic seizures before onset. We used DWT with Daubechies function for decomposing the signals and analyzing EEG recordings before onset and during the seizure for patients with epileptic seizures and with no epileptic seizures. To design the model, we used the predictive analysis of EEG signals, artificial feed-forward neural networks based on the Levenberg–Marquardt backpropagation optimization algorithm. In addition, we analyzed the covariance between biomedical signals (EEG, PPG, and EMG) to select the signals that can be used on predicting epileptic seizures. 

We can conclude that using the ANN with 150 neurons has an excellent performance in comparison with the ANN with ten neurons. However, this ANN analyzes requests an increased time in comparison with an ANN with a lower neuron number (e.g., ten neurons). Even if the use of an ANN with a large number of neurons gives more precision, it requires a very long time for data processing, and it is preferable to choose neural networks that provide an adequate solution about the issues regarding the accuracy of the outputs and the time allocated for processing [80].

The analysis of the covariance and correlation between signals allows the identification of biomedical signals that can be used in the predictive ANN applications for medical alert systems to send alerts if the regression at time *t* has a different value from the regression recorded in the analysis of signals taken from patients with no seizures activity [80].

The proposed methods showed promising results compared to other state-of-the-art methods. Our method opens new perspectives to the successful automatic detection of epileptic seizures before onset, enabling a real-time brain monitoring wearable system.

In the future, we plan to apply this method to epileptic signal detection on wearable devices. Our next research object is to develop a successful seizure forecasting model by analyzing, in addition, heart electrical activity (ECG).

## Figures and Tables

**Table 1:** ANN parameters.

Neurons No.	Input Data [Samples EEG3]	Output (Target) Data [Samples EEG1]	Train Set [Samples]	Test Set [Samples]	Validation Set [Samples]
10	Matrix 13 × 5120	Matrix 13 × 5120	3584	768	768
50	Matrix 13 × 5120	Matrix 13 × 5120	3584	768	768
100	Matrix 13 × 5120	Matrix 13 × 5120	3584	768	768
150	Matrix 13 × 5120	Matrix 13 × 5120	3584	768	768

**Table 2:** Value *R* = *f* (neural network neurons).

Neurons No.	R Training	R Validation	R Test	Processing Time [s]
10	0.57316	0.63855	0.49103	42
50	0.65267	0.60571	0.60182	745
100	0.85089	0.74129	0.82255	913
150	0.81819	0.77345	0.67324	2784

**Table 3:** Methodology description of recent state-of-the-art compared with our results.

Literature	Features Extraction Method	Learning Machine Method	Validation	Classification Accuracy
[69]				
Method 1	-	Convolutional neural network (CNN) with 3 layers	6-fold cross validation	83.8–95%
[70]				
Method 2	spectral and spatial features	SVM	-	96%
[71]				
Method 3	wavelet transform for decomposition	ANN and genetic algorithm	-	-
[72]				
Method 4	wavelet transform for decomposition	negative correlation learning (NCL) and a mixture of experts (ME)	25% of the train set was randomly selected for the validation set	96.92%
[73]				
Method 5	Multi-wavelet Transform	ANN	-	90%
[74]				
Method 6	-	pyramidal one-dimensional CNN (P-1D-CNN)	10-fold cross validation	99.1%
[75]				
Method 7	-	13-layer CNN	10-fold cross-validation	88.67%
[76]				
Method 8	DWT	SVM		96%
[77]				
Method 9	Minimum redundancy maximum relevance (mRMR), Principal component analysis (PCA)	SVM, k-nearest neighbors (k-NN), and discriminant analysis	Leave-one-out cross-validation	51% (SVM) 80% (k-nn with mRMR)
[78]				
Method 10	-	CNN	20-fold and 10-fold cross-validation	84.26%
[79]				
Method 11	-	U-Time—convolutional encoder-decoder network	5-fold cross-validation	-
Our work	DWT	ANN	15% of the samples were selected for the validation set	91.1%
